# Vibration of a Rotating Micro-Ring under Electrical Field Based on Inextensible Approximation

**DOI:** 10.3390/s18072044

**Published:** 2018-06-26

**Authors:** Tao Yu, Jiange Kou, Yuh-Chung Hu

**Affiliations:** 1 School of Mechatronics and Automobile Engineering, Yantai University, Yantai 264005, China; yutao@ytu.edu.cn (T.Y.); koujiange2018@gmail.com (J.K.); 2 Department of Mechanical and Electromechanical Engineering, National ILan University, Yilan City, Yilan County 260, Taiwan

**Keywords:** electrical field, electrostatics, MEMS, ring gyroscope, rotating ring, vibration

## Abstract

The problem of vibrations of rotating rings has been of interest for its wide applications in engineering, such as the vibratory ring gyroscopes. For the vibratory ring gyroscopes, the vibration of a micro ring is usually actuated and sensed by means of electrostatics. The analytical models of electrostatic microstructures are complicated due to their non-linear electromechanical coupling behavior. Therefore, this paper presents for the first time the free vibration of a rotating ring under uniform electrical field and the results will be helpful for extending our knowledge on the problem of vibrations of rotating rings, helping the design of vibratory ring gyroscopes, and inspiring the feasibilities of other engineering applications. An analytical model, based on thin-ring theory, is derived by means of energy method for a rotating ring under uniformly distributed electrical field. After that, the closed form solutions of the natural frequencies and modes are obtained by means of modal expansion method. Some valuable conclusions are made according to the results of the present analytical model. The electrical field causes not only an electrostatic force but also an equivalently negative electrical-stiffness. The equivalent negative electrical-stiffness will reduce either the natural frequencies or critical speeds of the rotating ring. It is known that the ring will buckle when its rotational speed equals its natural frequencies. The introduction of electrical field will further reduce the buckling speeds to a value less than the natural frequencies. The rotation effect will induce the so-called traveling modes, each one travels either in the same direction as the rotating ring or in the opposite direction with respect to stationary coordinate system. The electrical field will reduce the traveling velocities of the traveling modes.

## 1. Introduction

The problem of vibrations of rotating rings has been of interest for over a century due to its wide applications in engineering [1], e.g., tires [2,3], bearing [4], compliant gears [5], sensors [6], etc. Many scholars have continuously and systematically expanded our understanding of the behavior of rotating rings by studying the effects of rotatory inertia and shear, foundation stiffness, pre-stress or rotation, and the forced vibrations subjected to different types of loads, e.g., harmonic, periodic, distributed, traveling, etc. [1,2,3,7,8]. On the other hand, the technologies of microelectromechanical systems (MEMS) has been receiving much attention over the past two decades. MEMS are electromechanical integrated systems whose feature size of structures are within micro-scale. One of the applications of vibratory ring in MEMS is the vibratory ring gyroscopes. For the vibratory ring gyroscope, the vibration of a micro ring is usually actuated and sensed by means of electrostatics [9,10]. The electrostatics are commonly used in MEMS for actuating/sensing due to the advantages of rapid response, low power consumption, and being compatible with the standard fabrication process of Complementary-Metal-Oxide-Semiconductor (CMOS). The analytical models of electrostatic microstructures are complicated due to their non-linear electromechanical coupling behavior. One of the authors of this article, Hu, published a review article [11] to introduce the modeling of micro-devices adopting electrostatic actuating/sensing methods as well as the effects of non-ideal boundary conditions, fringing fields, pre-strain/-stress, and non-homogeneous structures. In 2004, Hu investigated the dynamical stability of a microstructure driven by alternative voltage for the first time [12]. Since then, many studies about the dynamical stability of electrostatic microstructures have been published continuously. As regards the vibration of micro rings, Hu, Chen, and Yu et al. investigated the forced response and stability of a micro-ring subjected to the concentrated electrostatic forces traveling around it along the radial direction [13,14]. After surveying many correlative studies, we found that there is no research on the free vibrations of a rotating ring under uniform electrical filed. Therefore, this paper presents for the first time the free vibration of a rotating ring under uniform electrical field and the results will be helpful for extending our knowledge on the problem of vibrations of rotating rings, helping the design of vibratory ring gyroscopes, and inspiring the feasibilities of other engineering applications.

The feature of rings in engineering applications is usually in the scope of thin ring, i.e., rings with the thickness-to-radius ratio inferior to 0.1 [7]. Furthermore, the diameter of the ring of a vibratory ring gyroscope is about several hundred micrometers while its thickness and the gap between the ring and the driving/sensing electrodes are only about several micrometers and one micrometer respectively [9,10,15]. Therefore, it is rational to model the rotating ring based on Euler–Bernoulli theory and uniform electrical field. Firstly, the authors derive the energy expressions of a rotating ring under a uniformly distributed electrical field around the ring along the radial direction. The total energy includes the translational kinetic energy and mechanical strain energy of the ring and the work done by the uniform electrical field. Then, the equations of motion are derived by means of Hamilton’s principle [16]. The operation of the ring gyroscope relies on the electrostatic actuation and capacitive sensing in the radial direction of the ring and on the first two flexural vibration modes and these modes are bending-dominant. Furthermore, the deformation of the ring is limited in the gap between the ring and the driving/sensing electrode and thus is in the scope of small deformation because its deformation is smaller than its thickness. Therefore, based on the above-mentioned bending-dominant modes and small deformation, the authors further linearize the electrostatic force term by means of truncating the non-linear higher-order terms of its Taylor series expansion and reduce the order of the system by means of inextensible approximation. After that, the closed-form solution of free vibration is obtained by means of modal expansion method. The effects of rotation and electrical field on the natural frequencies and modes of rotating ring are investigated as well.

## 2. Equations of Motion

**Figure 1:**
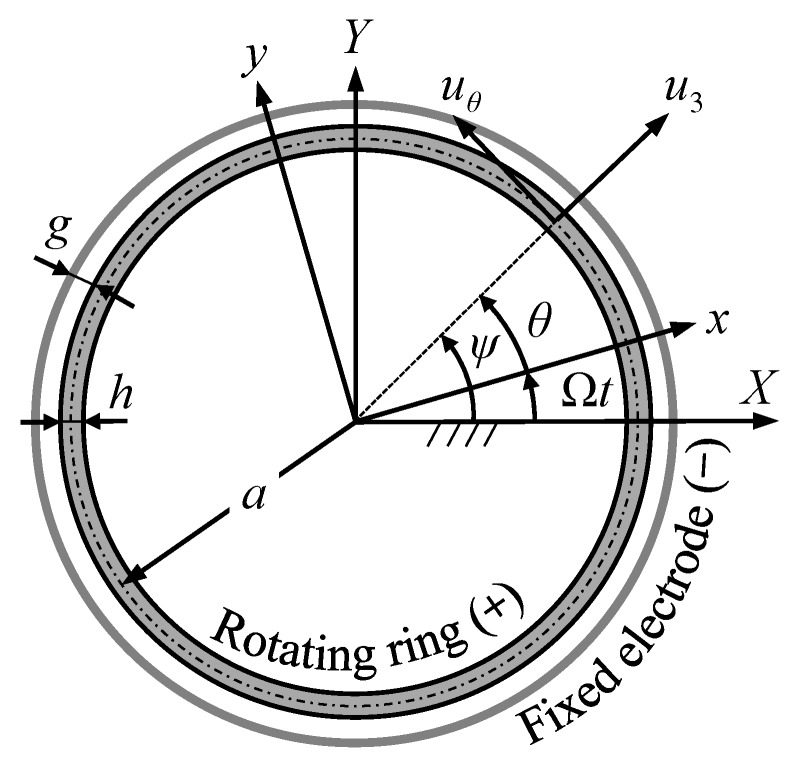
A flexural rotating ring surrounded by a circular fixed electrode, a uniformly distributed electrical field along the radial direction is made by applying an electrical potential difference between the ring and fixed electrode.

Consider a rotating ring surrounded by a circular fixed electrode (Figure 1). Both have the same width. An electrical potential difference (*V*) is made between the rotating ring and fixed electrode and thereby causes a uniformly distributed electrical field along the radial direction of the ring. According to thin ring theory [1], which is based on Euler–Bernoulli assumption, the plane sections remain plane after deformation and normal to the neutral surface, the shear deformation in transverse direction is negligible and the only significant strain is therefore in the circumferential direction. The rotary inertia is also negligible for thin rings. In Figure 1, *X*-*Y* is a stationary coordinate system while the coordinate system *x*-*y* rotates with the rotating ring at the angular speed of Ω. The circumferential and transverse deflections of the ring are denoted by $u_{\theta }$ and $u_{3}$, respectively; *θ* and *ψ* denote the angular position on the ring with respect to the rotating and stationary coordinate systems, respectively; *a*, *h*, and *b* denote the mean radius, thickness, and width of the ring, respectively; *g* is the initial gap between the ring and fixed electrode; and *t* is time. There is a potential difference, *V*, between the ring and fixed electrode. The mean radius of the ring is much larger than its thickness and the gap between the ring and fixed electrode is of the same order of magnitude of the ring’s thickness.

The total energy (*L*) of the entire system equals the sum of the kinetic energy (*T*) and strain energy (*U*) of the rotating ring and the work done (*W*) by the electrostatic force due to the uniformly distributed electrical field. For a mass element of the rotating ring with length *adθ*, its kinetic energy is

(1) $$dK=\frac{1}{2}\rho hbad\theta \vec{v}\cdot \vec{v},$$

where the velocity vector $\vec{v}$ is given by

(2) $$\vec{v}=a\Omega \;\vec{j}+({\dot{u}}_{3}\vec{i}+{\dot{u}}_{\theta }\vec{j})+\Omega \;\vec{k}\times (u_{3}\vec{i}+u_{\theta }\vec{j})=({\dot{u}}_{3}-u_{\theta }\Omega )\vec{i}+({\dot{u}}_{\theta }+u_{3}\Omega +a\Omega )\vec{j}.$$

Thus, the total kinetic energy of the ring per unit width is

(3) $$T=\frac{1}{2}\rho ha\int_{0}^{2\pi }\left[{\left({\dot{u}}_{3}-u_{\theta }\Omega \right)}^{2}+{\left({\dot{u}}_{\theta }+u_{3}\Omega +a\Omega \right)}^{2}\right]d\theta ,$$

The strain energy of the ring per unit width is given by [1]

(4) $$U=\int_{0}^{2\pi }\left[\frac{K}{2a^{2}}{\left(u_{\theta }^{\prime }+u_{3}\right)}^{2}+\frac{D}{2a^{4}}{\left(u_{\theta }^{\prime }-u_{3}^{\prime \prime }\right)}^{2}\right]ad\theta ,$$

where *D* = *Eh*^3^/12 is the bending stiffness and *K* = *Eh* is the membrane stiffness. As there is an electrical potential difference between the rotating ring and fixed electrode, the work done by the electrostatic force on the rotating ring per unit width is

(5) $$W=\int_{0}^{2\pi }\frac{1}{2}\frac{\varepsilon V^{2}}{g-u_{3}}ad\theta ,$$

where *ε* is the permittivity of the dielectric material between the rotating ring and fixed electrode.

Consider the motion of the rotating ring between two instants *t*_1_ and *t*_2_. Its configuration changes with time, tracing a path known as the true path. A slightly different path, known as the varied path, is obtained if at any given instant one allows a small variation in deflections (*δu_θ_* and *δu*_3_) with no associated change in time (*δt* = 0) and that the true and varied paths coincide at the two instants *t*_1_ and *t*_2_. Along the varied path, the variations of the associated energy expressions are given by

(6) $$\delta T=\rho ha\int_{0}^{2\pi }\left[\left({\dot{u}}_{\theta }+u_{3}\Omega +a\Omega \right)\Omega \delta u_{3}+\left({\dot{u}}_{3}-u_{\theta }\Omega \right)\delta {\dot{u}}_{3}-\left({\dot{u}}_{3}-u_{\theta }\Omega \right)\Omega \delta u_{\theta }+\left({\dot{u}}_{\theta }+u_{3}\Omega +a\Omega \right)\delta {\dot{u}}_{\theta }\right]d\theta ,$$

(7) $$\delta U=\int_{0}^{2\pi }\left\{\left[-\frac{D}{a^{4}}\left(u_{\theta }^{\prime \prime \prime }-u_{3}^{\prime \prime \prime \prime }\;\right)+\frac{K}{a^{2}}\left(u_{\theta }^{\prime }+u_{3}\right)\right]\delta u_{3}+\left[-\frac{D}{a^{4}}\left(u_{\theta }^{\prime \prime }-u_{3}^{\prime \prime \prime }\right)-\frac{K}{a^{2}}\left(u_{\theta }^{\prime \prime }+u_{3}^{\prime }\right)\right]\delta u_{\theta }\right\}ad\theta ,$$

(8) $$\delta W=\int_{0}^{2\pi }\frac{\varepsilon V^{2}}{2{\left(g-u_{3}\right)}^{2}}\delta u_{3}ad\theta .$$

Hamilton’s principle [16] states that the true path of a system between two specified states at two instants *t*_1_ and *t*_2_ renders the integral of the total energy variation in time stationary with respect to all possible varied paths, namely

(9) $$\int_{t_{0}}^{t_{1}}(\delta U-\delta T-\delta W)dt=0,$$

which gives

(10) $$\begin{array}{l} \int_{t_{9}}^{t_{1}}\int_{0}^{2\pi }\{\left[-\frac{D}{a^{4}}\left(u_{\theta }^{\prime \prime \prime }-u_{3}^{\prime \prime \prime \prime }\;\right)+\frac{K}{a^{2}}\left(u_{\theta }^{\prime }+u_{3}\right)+\rho h\left({\ddot{u}}_{3}-2\Omega {\dot{u}}_{\theta }-(a+u_{3})\Omega^{2}\right)-\frac{\varepsilon V^{2}}{2{\left(g-u_{3}\right)}^{2}}\right]\delta u_{3}\\ \;\;\;\;\;\;\;\;\;+\left[-\frac{D}{a^{4}}\left(u_{\theta }^{\prime \prime }-u_{3}^{\prime \prime \prime }\;\right)-\frac{K}{a^{2}}\left(u_{\theta }^{\prime \prime }+u_{3}^{\prime }\right)+\rho h\left({\ddot{u}}_{\theta }+2\Omega {\dot{u}}_{3}-u_{\theta }\Omega^{2}\right)\right]\delta u_{\theta }\}ad\theta dt=0 \end{array}.$$

The equation can be satisfied only if each of the double integral parts is 0 individually. Moreover, since the displacement variations are arbitrary, each integral equation can be satisfied only if the coefficients of the displacement variations are 0. Thus, setting the coefficients of the double integral to zero gives the following two equations of motion:

(11) $$\frac{D}{a^{4}}\left(u_{3}^{\prime \prime \prime \prime }-u_{\theta }^{\prime \prime \prime }\;\;\right)+\frac{K}{a^{2}}\left(u_{\theta }^{\prime }+u_{3}\right)+\rho h\left({\ddot{u}}_{3}-2\Omega {\dot{u}}_{\theta }-u_{3}\Omega^{2}\right)-\rho ha\Omega^{2}-\frac{\varepsilon V^{2}}{2{\left(g-u_{3}\right)}^{2}}=0,$$

(12) $$\frac{D}{a^{4}}\left(u_{3}^{\prime \prime \prime }-u_{\theta }^{\prime \prime }\right)-\frac{K}{a^{2}}\left(u_{\theta }^{\prime \prime }+u_{3}^{\prime }\right)+\rho h\left({\ddot{u}}_{\theta }+2\Omega {\dot{u}}_{3}-u_{\theta }\Omega^{2}\right)=0.$$

In Equation (11), the first term attributes to bending moment, the second term to membrane force, the third term to inertia and rotation effects, the fourth term to the centrifugal force, and the fifth term to the uniformly distributed electrical field along the radial direction. In Equation (12), the first term attributes to bending moment, the second term to membrane force, and the third term to inertia and rotation effects. If the electrical potential difference *V* is 0, then Equations (11) and (12) reduce to the equations of motion of a rotating ring; and if the rotating speed Ω is further 0, then further reduce to the equations of motion of non-rotating ring. In summary, the analytical model is based on thin ring theory, the plane section of the ring remains plane after deformation and normal to the neutral surface, the shear deformation in transverse direction is negligible and the only significant strain is therefore in the circumferential direction. The rotary inertia is also negligible for thin rings.

In practice, the mean radius of the ring is much larger than its thickness and the gap between the ring and fixed electrode is of the same order of magnitude of the ring’s thickness. Therefore, the deflection *u*_3_ is much smaller than the dimensions of the ring. Expanding the electrical-field term of Equation (11) by Taylor series with respect to the initial equilibrium position and, because the deflection *u*_3_ is much smaller than the dimensions of the ring in practice, neglecting the second- and higher-order terms based on the small deformation assumption gives

(13) $$-\frac{\varepsilon V^{2}}{2{\left(g-u_{3}\right)}^{2}}=-\frac{\varepsilon V^{2}}{2}\left[\frac{1}{g^{2}}+\frac{2}{g^{3}}u_{3}+\frac{3}{g^{4}}u_{3}^{2}+\frac{4}{g^{5}}u_{3}^{3}+\frac{5}{g^{6}}u_{3}^{4}+\cdots \right]\approx -\frac{\varepsilon V^{2}}{2}\left[\frac{1}{g^{2}}+\frac{2}{g^{3}}u_{3}\right].$$

Therefore, Equation (11) is linearized to be

(14) $$\frac{D}{a^{4}}\left(u_{3}^{\prime \prime \prime \prime }-u_{\theta }^{\prime \prime \prime }\;\;\right)+\frac{K}{a^{2}}\left(u_{\theta }^{\prime }+u_{3}\right)+\rho h\left({\ddot{u}}_{3}-2\Omega {\dot{u}}_{\theta }-u_{3}\Omega^{2}\right)-\frac{\varepsilon V^{2}}{g^{3}}u_{3}=\rho ha\Omega^{2}+\frac{\varepsilon V^{2}}{2g^{2}}.$$

Equation (14) depicts that, in addition to causing a uniformly distributed electrostatic force along radial direction, the uniformly distributed electrical field also causes an equivalently electrical stiffness, $-\varepsilon V^{2}/g^{3}$, along radial direction and thus alter the dynamical characteristic of the ring. It must be emphasized that the equivalently electrical stiffness is a negative value. On the other hand, in addition to causing a centrifugal force *ρha*Ω^2^, the Coriolis acceleration due to rotation further alter the dynamical characteristic of the rotating ring. The sum of $\rho ha\Omega^{2}+\varepsilon V^{2}/2g^{2}$ in Equation (14) causes an initially static equilibrium position of the ring.

Since there is no circumferential forcing, then the transverse (bending modes) vibration is predominant in the present case. Start with the linearized equations of motion, Equations (12) and (14), in terms of force and moment resultants:

(15) $$\frac{1}{a^{2}}\frac{\partial^{2}M_{\theta \theta }}{\partial \theta^{2}}-\frac{N_{\theta \theta }}{a}-\rho A\left(\frac{\partial^{2}u_{3}}{\partial t^{2}}-2\Omega \frac{\partial u_{\theta }}{\partial t}-\Omega^{2}u_{3}\right)+\frac{\varepsilon bV^{2}}{g^{3}}u_{3}=-\rho hab\Omega^{2}-\frac{\varepsilon bV^{2}}{2g^{2}},$$

(16) $$\frac{1}{a}\frac{\partial N_{\theta \theta }}{\partial \theta }+\frac{1}{a^{2}}\frac{\partial M_{\theta \theta }}{\partial \theta }-\rho A\left(\frac{\partial^{2}u_{\theta }}{\partial t^{2}}+2\Omega \frac{\partial u_{3}}{\partial t}-\Omega^{2}u_{\theta }\right)=0.$$

where *A* = *bh* is the cross-sectional area of the ring and *I* = *bh*^3^/12 is the area inertia moment of the cross-sectional area, and [1]

(17) $$M_{\theta \theta }=\frac{EI}{a^{2}}\left(\frac{\partial u_{\theta }}{\partial \theta }-\frac{\partial^{2}u_{3}}{\partial \theta^{2}}\right),\;N_{\theta \theta }=\frac{EA}{a}\left(\frac{\partial u_{\theta }}{\partial \theta }+u_{3}\right).$$

Solving Equation (15) for *N_θθ_* gives

(18) $$N_{\theta \theta }=\frac{1}{a}\frac{\partial^{2}M_{\theta \theta }}{\partial \theta^{2}}-\rho aA\left(\frac{\partial^{2}u_{3}}{\partial t^{2}}-2\frac{\partial u_{\theta }}{\partial t}\Omega -u_{3}\Omega^{2}\right)+\frac{\varepsilon abV^{2}}{g^{3}}u_{3}+\frac{\varepsilon abV^{2}}{2g^{2}}+\rho ha^{2}b\Omega^{2},$$

and substitute it in Equation (16),

(19) $$\frac{1}{a^{2}}\frac{\partial^{3}M_{\theta \theta }}{\partial \theta^{3}}+\frac{1}{a^{2}}\frac{\partial M_{\theta \theta }}{\partial \theta }-\rho A\left(\frac{\partial^{2}u_{\theta }}{\partial t^{2}}+2\Omega \frac{\partial u_{3}}{\partial t}-\Omega^{2}u_{\theta }\right)-\rho A\left(\frac{\partial^{3}u_{3}}{\partial t^{2}\partial \theta }-2\Omega \frac{\partial^{2}u_{\theta }}{\partial t\partial \theta }-\Omega^{2}\frac{\partial u_{3}}{\partial \theta }\right)+\frac{\varepsilon bV^{2}}{g^{3}}\frac{\partial u_{3}}{\partial \theta }=0.$$

By the inextensible approximation [1], namely the normal strain in circumferential direction of the neutral surface of the ring is zero:

(20) $$\varepsilon_{\theta \theta }^{0}=\frac{\partial u_{\theta }}{a\partial \theta }+\frac{u_{3}}{a}=0,$$

which implies that

(21) $$\frac{\partial u_{\theta }}{\partial \theta }=-u_{3}.$$

Applying the inextensible approximation in the relationship of moment and deflection, Equation (17), gives

(22) $$M_{\theta \theta }=-\frac{EI}{a^{2}}\left(u_{3}+\frac{\partial^{2}u_{3}}{\partial \theta^{2}}\right),$$

and substituting it in Equation (19) results in

(23) $$\frac{D}{a^{4}}\left(\frac{\partial^{6}u_{3}}{\partial \theta^{6}}+2\frac{\partial^{4}u_{3}}{\partial \theta^{4}}+\frac{\partial^{2}u_{3}}{\partial \theta^{2}}\right)-\frac{\varepsilon V^{2}}{g^{3}}\frac{\partial^{2}u_{3}}{\partial \theta^{2}}+\rho h\left[\frac{\partial^{4}u_{3}}{\partial \theta^{2}\partial t^{2}}+4\Omega \frac{\partial^{2}u_{3}}{\partial \theta \partial t}-\frac{\partial^{2}u_{3}}{\partial t^{2}}+\Omega^{2}\left(u_{3}-\frac{\partial^{2}u_{3}}{\partial \theta^{2}}\right)\right]=0.$$

Equation (23) is the equation of motion of a rotating ring under a uniformly distributed electrical field based on inextensible approximation.

## 3. Natural Frequencies

Since the geometrical periodicity of ring, let its transverse deflection function be

(24) $$u_{3}(\theta ,t)=A_{n}e^{j(n\theta +\omega_{n}t)}.$$

Substituting it into Equation (23) gives the equation of natural frequency

(25) $$\omega_{n}^{2}-\frac{4n\Omega }{\left(n^{2}+1\right)}\omega_{n}-\frac{n^{2}{\left(n^{2}-1\right)}^{2}}{\left(n^{2}+1\right)}\frac{D}{\rho ha^{4}}+\frac{n^{2}}{\left(n^{2}+1\right)}\frac{\varepsilon V^{2}}{\rho hg^{3}}+\Omega^{2}=0.$$

Then, one has two natural frequencies (*ω_n_*_1_ and *ω_n_*_2_) for every value of *n* ≥ 2:

(26) $$\omega_{n1}=\frac{2n}{n^{2}+1}\Omega -\sqrt{\frac{n^{2}{\left(n^{2}-1\right)}^{2}}{\left(n^{2}+1\right)}\frac{D}{\rho ha^{4}}-\frac{n^{2}}{\left(n^{2}+1\right)}\frac{\varepsilon V^{2}}{\rho hg^{3}}-\frac{{\left(n^{2}-1\right)}^{2}}{{\left(n^{2}+1\right)}^{2}}\Omega^{2}},$$

and

(27) $$\omega_{n2}=\frac{2n}{n^{2}+1}\Omega +\sqrt{\frac{n^{2}{\left(n^{2}-1\right)}^{2}}{\left(n^{2}+1\right)}\frac{D}{\rho ha^{4}}-\frac{n^{2}}{\left(n^{2}+1\right)}\frac{\varepsilon V^{2}}{\rho hg^{3}}-\frac{{\left(n^{2}-1\right)}^{2}}{{\left(n^{2}+1\right)}^{2}}\Omega^{2}}.$$

This is different from the case of non-rotating ring in which only one natural frequency for every value of *n*. The bifurcations of natural frequencies are resulted from the Coriolis acceleration. For the flexural modes (*n* ≥ 2), Equations (26) and (27) can be expressed as

(28) $$\frac{\omega_{nk}}{\omega_{fn}}=\frac{2n}{\left(n^{2}+1\right)}\frac{\Omega }{\omega_{fn}}\mp \sqrt{1-\frac{n^{2}}{\left(n^{2}+1\right)}{\left(\frac{\sqrt{\varepsilon V^{2}/\left(\rho hg^{3}\right)}}{\omega_{fn}}\right)}^{2}-\frac{{\left(n^{2}-1\right)}^{2}}{{\left(n^{2}+1\right)}^{2}}{\left(\frac{\Omega }{\omega_{fn}}\right)}^{2}},\;\mathrm{for}\;k=1,\;2$$

where $\omega_{fn}$ is the natural frequencies of a non-rotating ring (Ω = 0) in the absence of electric field (*V* = 0),

(29) $$\omega_{fn}=\sqrt{\frac{n^{2}{\left(n^{2}-1\right)}^{2}}{\left(n^{2}+1\right)}\frac{D}{\rho ha^{4}}}.$$

It should be mentioned that Equation (28) is not valid for *n* = 0 and 1 because of the inextensible approximation and rigid-body mode. The mode at *n* = 0 is sometimes called the breathing mode of the ring due to pure extension. At *n* = 1, the natural frequency, *ω_f_*_1_, of a non-rotating ring in the absence of electric field is zero. Thus, a flexural vibration still does not exist. One has to think of the ring as simply being displaced in a rigid body motion. The roots of the frequency equation, Equation (25), have three possibilities: two distinct roots of real numbers, two double roots of real numbers, and two complex conjugate roots. To ensure two distinct or double roots of real numbers, the terms in the square root symbol of Equation (28) must be equal or greater than zero, i.e.,

(30) $$\frac{{\left(\sqrt{\varepsilon V^{2}/\left(\rho hg^{3}\right)}/\omega_{fn}\right)}^{2}}{\left(n^{2}+1\right)/n^{2}}+\frac{{\left(\Omega /\omega_{fn}\right)}^{2}}{{\left(n^{2}+1\right)}^{2}/{\left(n^{2}-1\right)}^{2}}\leq 1,$$

otherwise two complex conjugate roots of the form α−jβ and α+jβ where the latter makes the vibration of ring decaying with time and the former makes it diverging, namely unstable.

**Figure 2:**
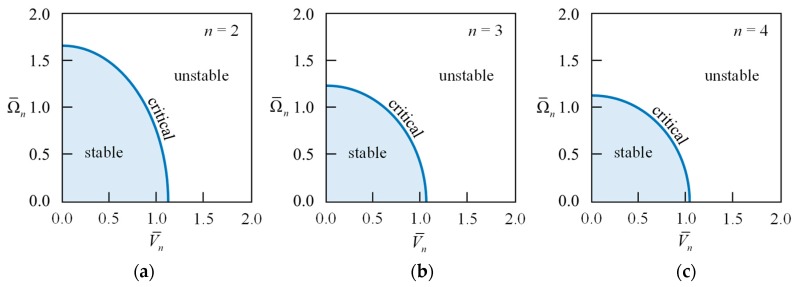
The stable regions, given by Equation (30), of the rotating ring under a uniformly distributed electrical field, wherein both abscissa and ordinate are normalized to be dimensionless rotational speed ${\bar{\Omega }}_{n}=\Omega /\omega_{fn}$ and dimensionless voltage ${\bar{V}}_{n}=\sqrt{\varepsilon V^{2}/\rho hg^{3}}/\omega_{fn}$. (**a**) *n* = 2; (**b**) *n* = 3; (**c**) *n* = 4.

Figure 2 visualizes Equation (30), wherein both abscissa and ordinate are normalized to be dimensionless rotational speed ${\bar{\Omega }}_{n}=\Omega /\omega_{fn}$ and dimensionless voltage ${\bar{V}}_{n}=\sqrt{\varepsilon V^{2}/\rho hg^{3}}/\omega_{fn}$. The stable region is a quarter ellipse, and as *n* gets larger, it approaches a quarter circle with a radius of unity. The rotational speeds on the boundary of the stable region are known as the critical speeds of the rotating ring. Due to the equivalent negative stiffness of the electrical field, the potential difference will reduce the critical speed. This is similar to the stiffness softened phenomenon by electrostatic force [12,13,14]. In the following, we discuss the vibration characteristics of the rotating ring under uniformly distributed electrical field only in the stable region.

**Figure 3:**
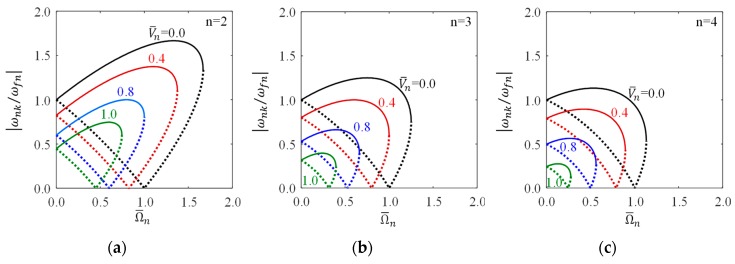
The bifurcation of natural frequencies due to rotation effect under different voltage, wherein the dashed curves are for $\omega_{n1}$ and solid curves for $\omega_{n2}$, ${\bar{\Omega }}_{n}=\Omega /\omega_{fn}$, and ${\bar{V}}_{n}=\sqrt{\varepsilon V^{2}/\rho hg^{3}}/\omega_{fn}$. (**a**) *n* = 2; (**b**) *n* = 3; (**c**) *n* = 4.

Figure 3 visualizes Equation (28) and illustrates the bifurcations of natural frequencies, wherein the abscissa and ordinate are respectively normalized to be dimensionless rotational speed, ${\bar{\Omega }}_{n}$, and the absolute value of dimensionless natural frequency, $\left|\omega_{nk}/\omega_{fn}\right|$, and the potential difference between the rotating ring and fixed electrode is also normalized to be dimensionless potential difference, ${\bar{V}}_{n}$. Two frequency curves, one dashed curve and one solid curve, are plotted for different value of ${\bar{V}}_{n}$, the dashed curve is for $\omega_{n1}$ and the solid curve for $\omega_{n2}$. The dashed and solid curves for every value of ${\bar{V}}_{n}$ have two intersections, one intersects at the ordinate and its value is given by Equation (31), and another intersection locates at the point satisfying Equation (32) which is exactly the point at the boundary of the stable region shown in Figure 2. The potential difference or electrical field reduces either the natural frequencies or the critical speeds of the rotating ring.

(31) $$\left|\frac{\omega_{nk}}{\omega_{fn}}\right|=\sqrt{1-\frac{n^{2}}{\left(n^{2}+1\right)}{\bar{V}}_{n}^{2}},$$

(32) $$\frac{{\left(\sqrt{\varepsilon V^{2}/\left(\rho hg^{3}\right)}/\omega_{fn}\right)}^{2}}{\left(n^{2}+1\right)/n^{2}}+\frac{{\left(\Omega /\omega_{fn}\right)}^{2}}{{\left(n^{2}+1\right)}^{2}/{\left(n^{2}-1\right)}^{2}}=1.$$

The dashed curves have a turning point at abscissa, namely a zero natural frequency occurs at the rotational speeds given by Equation (33). This is a buckling phenomenon induced by rotation effect. The rotating ring will buckle when the rotational speed equals its natural frequencies if *V* = 0. The introduction of electrical field, namely *V* ≠ 0, will further reduce the buckling speeds. In summary, the electrical field will reduce the natural frequencies, critical speeds, and buckling speeds of the rotating ring.

(33) $$\frac{\Omega }{\omega_{fn}}=\sqrt{1-\frac{n^{2}}{\left(n^{2}+1\right)}{\left(\frac{\sqrt{\varepsilon V^{2}/\left(\rho hg^{3}\right)}}{\omega_{fn}}\right)}^{2}}.$$

## 4. Natural Modes

Although Equation (24) defines the natural mode, it is instructive to use the stationary coordinate system, ψ=θ+Ωt, instead of *θ*, which gives

(34) $$u_{3k}(\theta ,t)=A_{nk}e^{jn\left[\psi -(\Omega -\omega_{nk}/n)t\right]},$$

for *k* = 1, 2. The natural modes are usually not functions of time. However, the modes defined in Equation (34) are functions of time because the rotation effect makes it difficult to separate the space and time coordinates in the usual manner, so the natural modes are time-dependent, which were named as traveling modes by Huang [2,3], as each one travels either in the same direction as the rotating ring or in the opposite direction to an observer who is not rotating with the ring, namely to an stationary coordinate. One can set *A_nk_* to be unity because its choice is arbitrary. To explain at what speeds the traveling modes rotate, we take the real part of Equation (34)

(35) $$\cos n\left[\psi -(\Omega -\omega_{nk}/n)t\right].$$

We set Equation (35) to its maximum possible value, unity, to see at what speeds the mode antinodes rotate:

(36) $$\cos n\left[\psi_{\max}-(\Omega -\omega_{nk}/n)t\right]=1.$$

This gives

(37) $$\psi_{\max}=2m\pi /n+\left(\Omega -\omega_{nk}/n\right)t,$$

where *m* = 0, 1, 2, 3, etc. Therefore, the speeds of the traveling modes with respect to a stationary coordinate are given by

(38) $${\dot{\psi }}_{\max1}=\Omega -\frac{\omega_{n1}}{n}=\frac{n^{2}-1}{n^{2}+1}\Omega +\frac{1}{n}\sqrt{\frac{n^{2}{\left(n^{2}-1\right)}^{2}}{\left(n^{2}+1\right)}\frac{D}{\rho ha^{4}}-\frac{n^{2}}{n^{2}+1}\frac{\varepsilon V^{2}}{\rho hg^{3}}-\frac{{\left(n^{2}-1\right)}^{2}}{{\left(n^{2}+1\right)}^{2}}\Omega^{2}},$$

and

(39) $${\dot{\psi }}_{\max2}=\Omega -\frac{\omega_{n2}}{n}=\frac{n^{2}-1}{n^{2}+1}\Omega -\frac{1}{n}\sqrt{\frac{n^{2}{\left(n^{2}-1\right)}^{2}}{\left(n^{2}+1\right)}\frac{D}{\rho ha^{4}}-\frac{n^{2}}{n^{2}+1}\frac{\varepsilon V^{2}}{\rho hg^{3}}-\frac{{\left(n^{2}-1\right)}^{2}}{{\left(n^{2}+1\right)}^{2}}\Omega^{2}}.$$

For the flexural modes (*n* ≥ 2), Equations (38) and (39) can be expressed as

(40) $$\frac{{\dot{\psi }}_{\max k}}{\omega_{fn}}=\frac{n^{2}-1}{n^{2}+1}\left(\frac{\Omega }{\omega_{fn}}\right)\pm \frac{1}{n}\sqrt{1-\frac{n^{2}}{n^{2}+1}{\left(\frac{\sqrt{\varepsilon V^{2}/\left(\rho hg^{3}\right)}}{\omega_{fn}}\right)}^{2}-\frac{{\left(n^{2}-1\right)}^{2}}{{\left(n^{2}+1\right)}^{2}}{\left(\frac{\Omega }{\omega_{fn}}\right)}^{2}},$$

for *k* = 1, 2, wherein $\omega_{fn}$ is the natural frequencies of a non-rotating ring (Ω = 0) in the absence of electric field (*V* = 0) and is given by Equation (29).

The modes become stationary if ${\dot{\psi }}_{\max}=\Omega -\omega_{nk}/n=0$; substituting this into Equation (40) gives

(41) $$\frac{{\left(\Omega /\omega_{fn}\right)}^{2}}{\left(n^{2}+1\right)/{\left(n^{2}-1\right)}^{2}}+\frac{{\left(\sqrt{\varepsilon V^{2}/\left(\rho hg^{3}\right)}/\omega_{fn}\right)}^{2}}{\left(n^{2}+1\right)/n^{2}}=1.$$

This means that, if the relationship is satisfied, the mode does not rotate but appears as a stationary distortion of the ring to an observer who is not rotating with the ring and the corresponding stationary modes are given by

(42) cosnψ.

**Figure 4:**
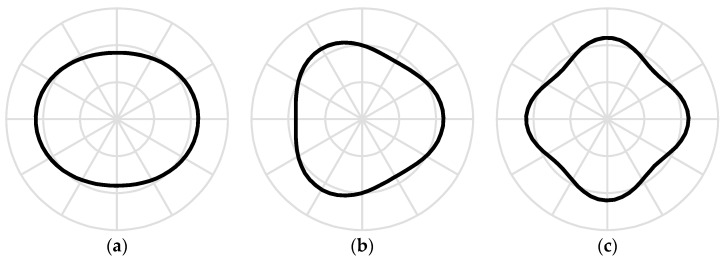
The stationary modes when Ω and *V* satisfy Equation (40). (**a**) *n* = 2; (**b**) *n* = 3; (**c**) *n* = 4.

Figure 4 shows the first three stationary flexural modes of the rotating ring. If ${\dot{\psi }}_{\max}=\Omega -\omega_{nk}/n>0$, wherein the ring’s rotational speed and the potential difference satisfy the relationship of Equation (43), then the mode antinodes lag behind the rotational speed Ω of the ring.

(43) $$\frac{{\left(\Omega /\omega_{fn}\right)}^{2}}{\left(n^{2}+1\right)/{\left(n^{2}-1\right)}^{2}}+\frac{{\left(\sqrt{\varepsilon V^{2}/\left(\rho hg^{3}\right)}/\omega_{fn}\right)}^{2}}{\left(n^{2}+1\right)/n^{2}}>1.$$

**Figure 5:**
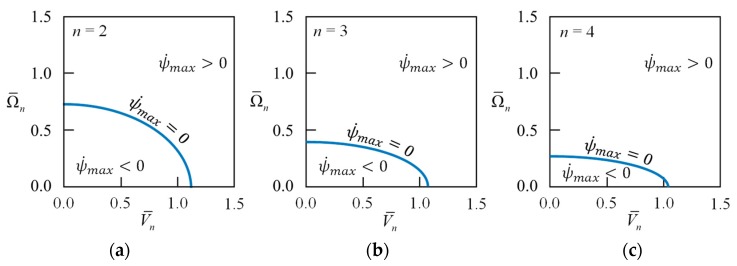
The signs (directions) of the speed of traveling modes, wherein both abscissa and ordinate are normalized to be dimensionless voltage, ${\bar{V}}_{n}=\sqrt{\varepsilon V^{2}/\left(\rho hg^{3}\right)}/\omega_{fn}$, and dimensionless rotational speed of ring, ${\bar{\Omega }}_{n}=\Omega /\omega_{fn}$. (**a**) *n* = 2; (**b**) *n* = 3; (**c**) *n* = 4.

If ${\dot{\psi }}_{\max}=\Omega -\omega_{nk}/n<0$, wherein the ring’s rotational speed and the potential difference satisfy the relationship of Equation (44), then the mode antinodes rotate in the direction opposite to the rotational speed Ω of ring. Figure 5 visualizes Equations (41), (43), and (44), wherein both abscissa and ordinate are normalized to be dimensionless rotational speed ${\bar{\Omega }}_{n}$ and voltage ${\bar{V}}_{n}$. The blue curve, satisfying Equation (41), is a quarter-ellipse and approaches to a horizontal line from the origin to ${\bar{V}}_{n}=1.0$ as *n* getting large. This means that the higher-order modes will travel in the same direction of the rotating ring but lag behind the rotational speed Ω of the ring.

(44) $$\frac{{\left(\Omega /\omega_{fn}\right)}^{2}}{\left(n^{2}+1\right)/{\left(n^{2}-1\right)}^{2}}+\frac{{\left(\sqrt{\varepsilon V^{2}/\left(\rho hg^{3}\right)}/\omega_{fn}\right)}^{2}}{\left(n^{2}+1\right)/n^{2}}<1.$$

For $\Omega -\omega_{nk}/n\neq 0$, there are two traveling modes corresponding to every value of *n*, namely the forward and backward traveling modes:

(45) $$\cos n(\psi -{\dot{\psi }}_{\max1}t),\;\cos n(\psi -{\dot{\psi }}_{\max2}t),$$

where the rotational speeds of the two traveling modes are given by

(46) $$\frac{{\dot{\psi }}_{\max1}}{\omega_{fn}}=\frac{n^{2}-1}{n^{2}+1}\frac{\Omega }{\omega_{fn}}+\frac{1}{n}\sqrt{1-\frac{n^{2}}{n^{2}+1}{\left(\frac{\sqrt{\varepsilon V^{2}/\rho hg^{3}}}{\omega_{fn}}\right)}^{2}-{\left(\frac{n^{2}-1}{n^{2}+1}\right)}^{2}{\left(\frac{\Omega }{\omega_{fn}}\right)}^{2}},$$

and

(47) $$\frac{{\dot{\psi }}_{\max2}}{\omega_{fn}}=\frac{n^{2}-1}{n^{2}+1}\frac{\Omega }{\omega_{fn}}-\frac{1}{n}\sqrt{1-\frac{n^{2}}{n^{2}+1}{\left(\frac{\sqrt{\varepsilon V^{2}/\rho hg^{3}}}{\omega_{fn}}\right)}^{2}-{\left(\frac{n^{2}-1}{n^{2}+1}\right)}^{2}{\left(\frac{\Omega }{\omega_{fn}}\right)}^{2}}.$$

**Figure 6:**
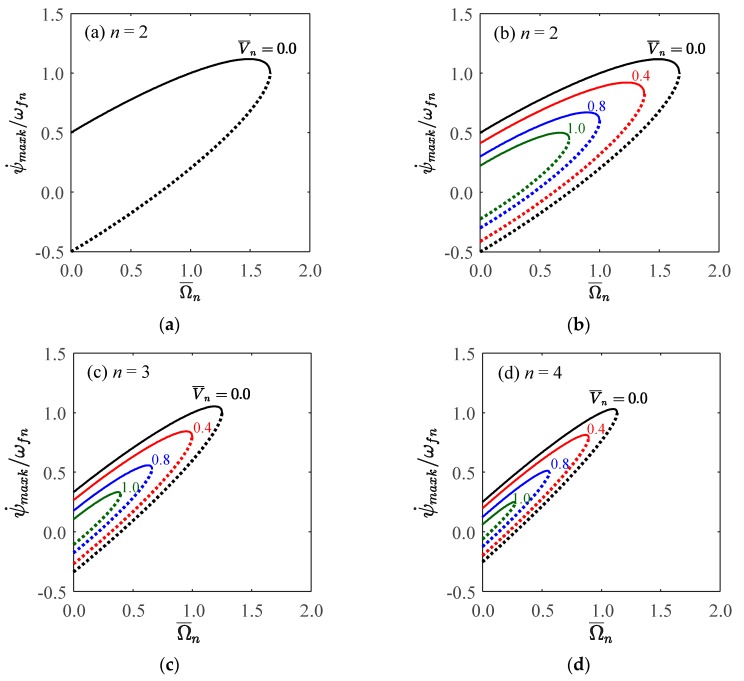
The rotational speeds of the forward (solid lines) and backward (dashed lines) traveling modes, where ${\bar{\Omega }}_{n}=\Omega /\omega_{fn}$ and ${\bar{V}}_{n}=\sqrt{\varepsilon V^{2}/\left(\rho hg^{3}\right)}/\omega_{fn}$. (**a**) *n* = 2 and ${\bar{V}}_{n}$ = 0; (**b**) *n* = 2; (**c**) *n* = 3; (**d**) *n* = 4.

**Figure 7:**
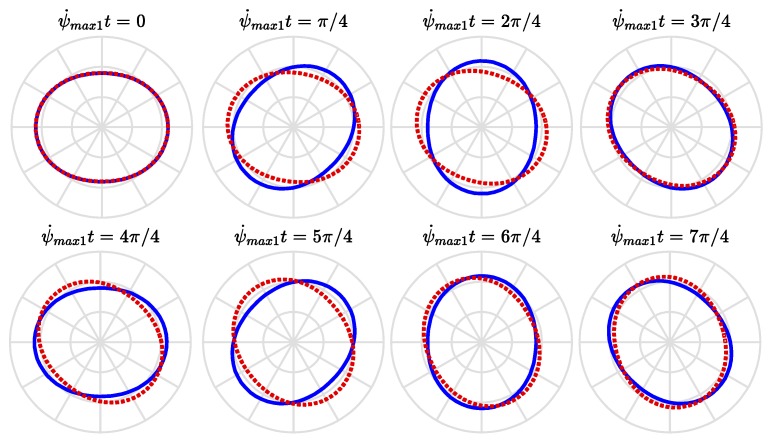
The traveling modes of *n* = 2 at various instance of time as viewed by a stationary observer.

**Figure 8:**
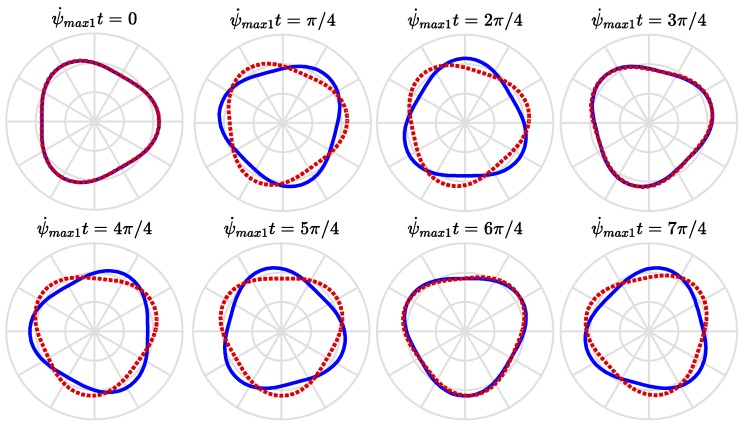
The traveling modes of *n* = 3 at various instance of time as viewed by a stationary observer.

Figure 6 visualizes the velocities of the traveling modes, Equations (46) and (47), wherein the abscissa and ordinate are normalized to be the dimensionless traveling speed of modes, ${\dot{\psi }}_{maxk}/\omega_{fn}$, and the dimensionless rotational speed of ring, ${\bar{\Omega }}_{n}$. The solid lines illustrate the velocities of the forward traveling modes while the dashed lines illustrate those of the backward traveling modes. It illustrates the effects of the potential difference and the ring’s rotational speed on the velocities of the traveling modes. The rotation effect of ring will make the speeds (the magnitudes of velocities) of the forward and backward traveling modes different. The forward traveling mode travels in the same direction of the rotating ring and its speed increases with the rotational speed of the ring. The backward traveling mode travels initially in the opposite direction of the rotating ring but its speed decreases with the rotational speed of the ring and becomes stationary when the rotational speed of the ring reaches at the value satisfying Equation (41); then, it turns to the same direction of the rotating ring and its speed increases with the rotational speed of the ring. The potential difference will reduce the traveling velocities of the forward and backward traveling modes. Figure 7 and Figure 8 show the traveling modes along the ring of *n* equal to 2 and 3, respectively. It is seen that the backward (clockwise) and forward (counterclockwise) modes travel at different speeds with respect to stationary coordinate system.

## 5. Conclusions

This paper derives an analytical model for a rotating ring under the uniformly distributed electrostatic field along the radial direction. By setting the potential difference between the ring and fixed electrode to zero, namely no electrical field, the present analytical model can be reduced to the well-known thin rotating ring model. It can also be further reduced to the well-known thin non-rotating ring model by setting both the potential difference and rotational speed to zero. The closed form solutions of the natural frequencies and modes of a rotating ring under uniform electrical field are obtained in this paper. Some conclusions are made according to the results of the present analytical model. The electrical field causes not only an electrostatic force but also an equivalently negative electrical-stiffness. Due to the equivalent negative stiffness, the electrical field will reduce either the natural frequencies or critical speeds of the rotating ring. For a rotating ring without the action of electrical field, it will buckle when the rotational speed equals its natural frequencies. The introduction of electrical field will further reduce the buckling speeds. The rotation effect will induce the so-called traveling modes, each one traveling either in the same direction as the rotating ring or in the opposite direction with respect to stationary coordinate system. The electrical field will reduce the traveling velocities of the traveling modes.
